# 2-Acetyl­amino-1,3,4,6-tetra-*O*-(tri­methyl­silyl)-2-de­oxy-α-d-gluco­pyran­ose

**DOI:** 10.1107/S160053681301266X

**Published:** 2013-05-18

**Authors:** Zhao-Dong Cheng, Yan-Li Cui, Jian-Wei Mao

**Affiliations:** a Department of Chemistry, Zhejiang University, Hangzhou, Zhejiang 310027, People’s Republic of China; b Zhejiang Provincial Key Lab for Chem. & Bio. Processing Technology of Farm Produce, Hangzhou, Zhejiang 310027, People’s Republic of China

## Abstract

The title compound, C_20_H_47_NO_6_Si_4_, was synthesized by per-*O*-tri­methyl­silylation of *N*-acetyl-d-glucosa­mine using chloro­tri­methyl­silane in the presence of hexa­methyl­disiloxane. The tri­methyl­silyl group and acetamido group are located on the same side of the pyran ring, showing an α-configuration glycoside. One of the tri­methyl­silyl groups is disordered over two orientations, with site-occupancy factors of 0.625 (9) and 0.375 (9). In the crystal, N—H⋯O hydrogen bonds link the mol­ecules into supra­molecular chains along the *a-*axis direction.

## Related literature

For background to the title compound, see: Augé *et al.* (1985 ▶); Ronnow *et al.* (1994 ▶); Du & Gervais-Hague (2005 ▶); Wang *et al.* (2007 ▶); Witschi & Gervais-Hague (2010 ▶). For related structures, see: Odinokov *et al.* (2002 ▶); Hu *et al.* (2011 ▶). For the synthesis, see: Loganathan & Trivedi (1987 ▶); Jervis *et al.* (2010 ▶).
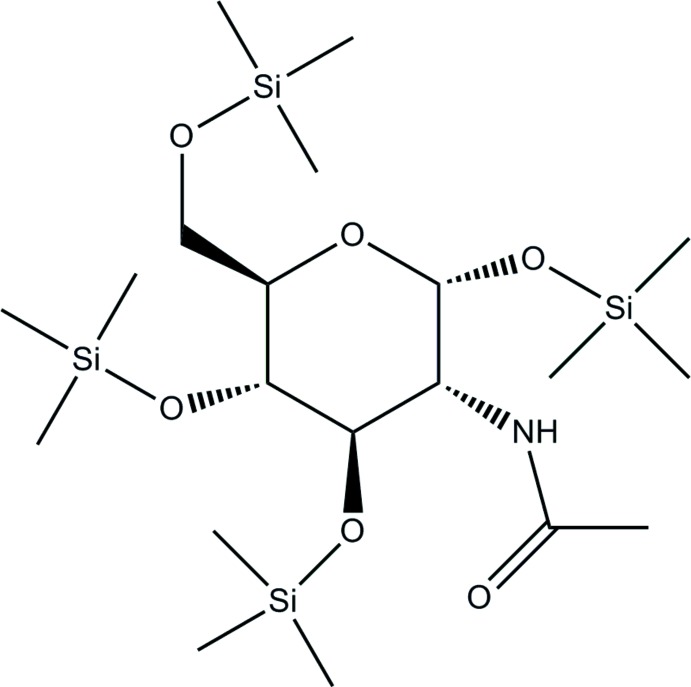


## Experimental

### 

#### Crystal data

C_20_H_47_NO_6_Si_4_
*M* *_r_* = 509.95
Orthorhombic, 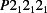

*a* = 9.4500 (7) Å
*b* = 12.8824 (9) Å
*c* = 27.295 (3) Å
*V* = 3322.9 (5) Å^3^
*Z* = 4
Mo *K*α radiation
μ = 0.21 mm^−1^
*T* = 293 K
0.38 × 0.20 × 0.19 mm

#### Data collection

Agilent Xcalibur (Atlas, Gemini ultra) diffractometer
Absorption correction: multi-scan (*CrysAlis PRO*; Agilent, 2011 ▶) *T*_min_ = 0.926, *T*_max_ = 0.962
9896 measured reflections
3438 independent reflections
2105 reflections with *I* > 2σ(*I*)
*R*_int_ = 0.046

#### Refinement

*R*[*F* ^2^ > 2σ(*F* ^2^)] = 0.055
*wR*(*F* ^2^) = 0.149
*S* = 1.01
3438 reflections
309 parameters
183 restraints
H-atom parameters constrained
Δρ_max_ = 0.26 e Å^−3^
Δρ_min_ = −0.22 e Å^−3^

### 

Data collection: *CrysAlis PRO* (Agilent, 2011 ▶); cell refinement: *CrysAlis PRO*; data reduction: *CrysAlis PRO*; program(s) used to solve structure: *SHELXS97* (Sheldrick, 2008 ▶); program(s) used to refine structure: *SHELXL97* (Sheldrick, 2008 ▶); molecular graphics: *OLEX2* (Dolomanov *et al.*, 2009 ▶); software used to prepare material for publication: *OLEX2*.

## Supplementary Material

Crystal structure: contains datablock(s) I, global. DOI: 10.1107/S160053681301266X/zq2199sup1.cif

Structure factors: contains datablock(s) I. DOI: 10.1107/S160053681301266X/zq2199Isup2.hkl

Additional supplementary materials:  crystallographic information; 3D view; checkCIF report

## Figures and Tables

**Table 1:** Hydrogen-bond geometry (Å, °)

*D*—H⋯*A*	*D*—H	H⋯*A*	*D*⋯*A*	*D*—H⋯*A*
N1—H1⋯O3^i^	0.86	2.03	2.855 (4)	160

Symmetry code: (i) 
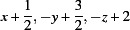
.

**Fig. 1.**
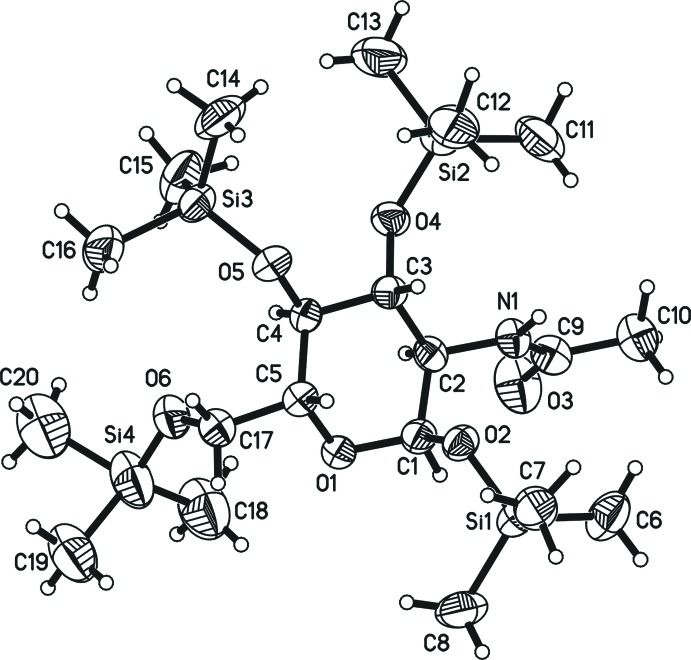
The molecular structure of the title compound with 30% probability displacement ellipsoids.

**Fig. 2.**
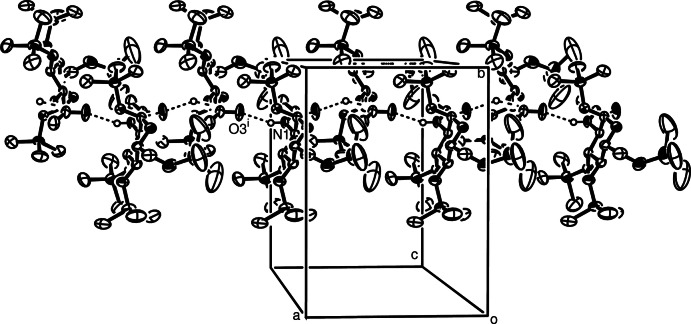
Crystal packing of the title compound showing N—H···O hydrogen bonds.

## supplementary crystallographic information

### Comment

Per-O-trimethylsilylated glycopyranose was an useful intermediate for the construction of oligosaccharides and glycoconjugates (Du & Gervais-Hague, 2005). Per-trimethylsilylation of unprotected sugar could increase its solubility in organic solvents, and made selective acetylation available (Witschi *et al.*, 2010). Hu *et al.* have developed an one-pot α-glycoside method which also used per-O-trimethylsilylated glycosides as starting materials (Hu *et al.*, 2011; Wang *et al.*, 2007). Currently we have applied considerable effort towards the construction of α-glycosides (Augé *et al.*, 1985; Ronnow *et al.*, 1994; Jervis *et al.*, 2010). We have synthesized the title compound and report its crystal structure herein (for related structures, see: Odinokov *et al.*, 2002; Hu *et al.*, 2011).

The molecular structure of the title compound is shown in Fig. 1. In the molecule, the trimethylsilyl group and acetamido group are located on the same side of the pyran ring, showing α configuration glycoside. One of the trimethylsilyl group is disordered over two orientations with site-occupancy factors of 0.625 (9) and 0.375 (9). In the crystal structure, weak intermolecular N—H···O hydrogen bonds link the molecules into supramolecular chains along the *a* axis in the crystal (Fig. 2).

### Experimental

To a solution of N-acetyl-D-glucosamine (1.0 g, 4.52 mmol) in pyridine (10 mL), hexamethyldisiloxane (HMDS) (8.0 mL, 38.90 mmol) and chlorotrimethylsilane (TMSCl) (4.0 mL, 31.64 mmol) were added sequentially. The solution was stirred at 353 K under a nitrogen atmosphere for 2 hours. After cooling to rt the mixture was poured into ice-water and extracted with hexane. The organic layers were washed with brine, dried with MgSO_4_, filtered, and concentrated in vacuum to furnish the crude product. The residue was purified by silica gel chromatography (petroether/ethyl acetate = 15:1) to afford the title compound. The crystal suitable for X-ray data collection was obtained by slow evaporation from a methanol solution (Jervis *et al.*, 2010; Loganathan *et al.*, 1987).

### Refinement

One of the trimethylsilyl group is disordered over two orientations with site-occupancy factors of 0.625 (9) and 0.375 (9). Some restraints and constraints had to be used to correct the geometry of the disordered components and the thermal parameters of the corresponding atoms. All H atoms were placed in geometrically idealized positions (C—H = 0.93–0.97Å) and constrained to ride on their parent atoms, with *U*_iso_(H) = 1.5*U*_eq_(C) for the methyl H atoms, and with *U*_iso_(H) = 1.2*U*_eq_(C) for the remaining H atoms.

### Crystal data

**Table:**

C_20_H_47_NO_6_Si_4_	*F*(000) = 1112
*M**_r_* = 509.95	*D*_x_ = 1.019 Mg m^−^^3^
Orthorhombic, *P*2_1_2_1_2_1_	Mo *K*α radiation, λ = 0.71073 Å
Hall symbol: P 2ac 2ab	Cell parameters from 1672 reflections
*a* = 9.4500 (7) Å	θ = 3.2–29.6°
*b* = 12.8824 (9) Å	µ = 0.21 mm^−^^1^
*c* = 27.295 (3) Å	*T* = 293 K
*V* = 3322.9 (5) Å^3^	Needle, colourless
*Z* = 4	0.38 × 0.20 × 0.19 mm

### Data collection

**Table:**

Agilent Xcalibur (Atlas, Gemini ultra) diffractometer	3438 independent reflections
Radiation source: fine-focus sealed tube	2105 reflections with *I* > 2σ(*I*)
Graphite monochromator	*R*_int_ = 0.046
Detector resolution: 10.3592 pixels mm^-1^	θ_max_ = 25.4°, θ_min_ = 3.2°
ω scans	*h* = −11→10
Absorption correction: multi-scan (*CrysAlis PRO*; Agilent, 2011)	*k* = −12→15
*T*_min_ = 0.926, *T*_max_ = 0.962	*l* = −30→32
9896 measured reflections	

### Refinement

**Table:**

Refinement on *F*^2^	Primary atom site location: structure-invariant direct methods
Least-squares matrix: full	Secondary atom site location: difference Fourier map
*R*[*F*^2^ > 2σ(*F*^2^)] = 0.055	Hydrogen site location: inferred from neighbouring sites
*wR*(*F*^2^) = 0.149	H-atom parameters constrained
*S* = 1.01	*w* = 1/[σ^2^(*F*_o_^2^) + (0.0744*P*)^2^ + 0.3973*P*] where *P* = (*F*_o_^2^ + 2*F*_c_^2^)/3
3438 reflections	(Δ/σ)_max_ < 0.001
309 parameters	Δρ_max_ = 0.26 e Å^−^^3^
183 restraints	Δρ_min_ = −0.22 e Å^−^^3^

### Special details

**Table:**

Geometry. All esds (except the esd in the dihedral angle between two l.s. planes) are estimated using the full covariance matrix. The cell esds are taken into account individually in the estimation of esds in distances, angles and torsion angles; correlations between esds in cell parameters are only used when they are defined by crystal symmetry. An approximate (isotropic) treatment of cell esds is used for estimating esds involving l.s. planes.
Refinement. Refinement of F^2^ against ALL reflections. The weighted R-factor wR and goodness of fit S are based on F^2^, conventional R-factors R are based on F, with F set to zero for negative F^2^. The threshold expression of F^2^ > 2sigma(F^2^) is used only for calculating R-factors(gt) etc. and is not relevant to the choice of reflections for refinement. R-factors based on F^2^ are statistically about twice as large as those based on F, and R- factors based on ALL data will be even larger.

### Fractional atomic coordinates and isotropic or equivalent isotropic displacement parameters (Å^2^)

**Table:**

	*x*	*y*	*z*	*U*_iso_*/*U*_eq_	Occ. (<1)
Si1	1.01789 (16)	0.89841 (11)	0.87673 (6)	0.0716 (4)	
Si2	1.03616 (19)	0.43173 (13)	0.99519 (7)	0.0911 (6)	
Si3	0.9320 (2)	0.31145 (12)	0.84396 (7)	0.0842 (5)	
Si4	0.5209 (19)	0.5532 (8)	0.7757 (5)	0.1318 (13)	0.625 (9)
O1	0.7944 (3)	0.6870 (3)	0.85017 (13)	0.0726 (9)	
O2	0.9849 (3)	0.7739 (2)	0.88456 (12)	0.0662 (9)	
O3	0.7055 (3)	0.7511 (5)	1.00819 (18)	0.1157 (16)	
O4	0.9211 (3)	0.4785 (3)	0.95578 (13)	0.0731 (10)	
O5	0.9704 (4)	0.4351 (3)	0.85373 (13)	0.0751 (9)	
O6	0.6763 (5)	0.5183 (4)	0.79227 (17)	0.1117 (16)	
N1	0.9130 (3)	0.7012 (3)	0.97859 (14)	0.0604 (11)	
H1	1.0030	0.6991	0.9831	0.072*	
C1	0.8492 (5)	0.7328 (4)	0.8930 (2)	0.0646 (13)	
H1A	0.7863	0.7895	0.9029	0.077*	
C2	0.8562 (4)	0.6535 (4)	0.93482 (18)	0.0581 (12)	
H2	0.7590	0.6320	0.9421	0.070*	
C3	0.9380 (4)	0.5563 (4)	0.91906 (17)	0.0593 (12)	
H3	1.0386	0.5733	0.9156	0.071*	
C4	0.8818 (5)	0.5161 (4)	0.87110 (18)	0.0612 (12)	
H4	0.7863	0.4886	0.8762	0.073*	
C5	0.8759 (5)	0.6012 (4)	0.83238 (19)	0.0690 (14)	
H5	0.9723	0.6250	0.8253	0.083*	
C6	1.0050 (8)	0.9663 (5)	0.9361 (2)	0.113 (2)	
H6A	0.9109	0.9591	0.9489	0.170*	
H6B	1.0262	1.0386	0.9316	0.170*	
H6C	1.0714	0.9366	0.9588	0.170*	
C7	1.1984 (6)	0.9046 (5)	0.8523 (2)	0.0970 (19)	
H7A	1.2648	0.8907	0.8781	0.146*	
H7B	1.2156	0.9726	0.8392	0.146*	
H7C	1.2093	0.8538	0.8269	0.146*	
C8	0.8876 (7)	0.9521 (5)	0.8333 (3)	0.113 (2)	
H8A	0.8682	0.9019	0.8082	0.169*	
H8B	0.9251	1.0140	0.8186	0.169*	
H8C	0.8017	0.9684	0.8504	0.169*	
C9	0.8345 (5)	0.7478 (5)	1.0119 (2)	0.0715 (15)	
C10	0.9086 (6)	0.7965 (5)	1.0546 (2)	0.0879 (17)	
H10A	0.9505	0.8610	1.0446	0.132*	
H10B	0.9812	0.7505	1.0662	0.132*	
H10C	0.8417	0.8092	1.0804	0.132*	
C11	1.0461 (9)	0.5095 (7)	1.0526 (2)	0.140 (3)	
H11A	1.0800	0.5780	1.0452	0.209*	
H11B	1.1097	0.4765	1.0752	0.209*	
H11C	0.9537	0.5141	1.0671	0.209*	
C12	1.2153 (7)	0.4319 (6)	0.9680 (3)	0.129 (3)	
H12A	1.2113	0.4030	0.9356	0.194*	
H12B	1.2776	0.3909	0.9880	0.194*	
H12C	1.2500	0.5018	0.9663	0.194*	
C13	0.9806 (11)	0.2963 (6)	1.0071 (4)	0.163 (4)	
H13A	0.8920	0.2963	1.0243	0.245*	
H13B	1.0511	0.2621	1.0266	0.245*	
H13C	0.9698	0.2603	0.9765	0.245*	
C14	1.0962 (9)	0.2396 (5)	0.8588 (3)	0.132 (3)	
H14A	1.1755	0.2733	0.8436	0.198*	
H14B	1.0888	0.1697	0.8468	0.198*	
H14C	1.1094	0.2384	0.8937	0.198*	
C15	0.7800 (8)	0.2703 (6)	0.8809 (3)	0.124 (3)	
H15A	0.7923	0.2935	0.9140	0.185*	
H15B	0.7732	0.1959	0.8803	0.185*	
H15C	0.6950	0.2998	0.8676	0.185*	
C16	0.8882 (11)	0.2878 (5)	0.7775 (3)	0.140 (3)	
H16A	0.8026	0.3239	0.7692	0.210*	
H16B	0.8754	0.2147	0.7721	0.210*	
H16C	0.9642	0.3126	0.7573	0.210*	
C17	0.8067 (7)	0.5670 (5)	0.7852 (2)	0.0922 (18)	
H17A	0.7933	0.6271	0.7643	0.111*	
H17B	0.8697	0.5195	0.7683	0.111*	
C18A	0.4994 (16)	0.7084 (13)	0.7939 (6)	0.161 (3)	0.625 (9)
H18A	0.5411	0.7212	0.8254	0.242*	0.625 (9)
H18B	0.4013	0.7276	0.7946	0.242*	0.625 (9)
H18C	0.5476	0.7488	0.7695	0.242*	0.625 (9)
C19	0.5118 (19)	0.5859 (15)	0.7098 (5)	0.151 (3)	0.625 (9)
H19A	0.5719	0.6443	0.7032	0.226*	0.625 (9)
H19B	0.4161	0.6028	0.7012	0.226*	0.625 (9)
H19C	0.5428	0.5275	0.6908	0.226*	0.625 (9)
C20A	0.3849 (15)	0.4802 (14)	0.8078 (6)	0.170 (3)	0.625 (9)
H20A	0.3810	0.4162	0.7898	0.255*	0.625 (9)
H20B	0.2932	0.5124	0.8076	0.255*	0.625 (9)
H20C	0.4128	0.4665	0.8410	0.255*	0.625 (9)
Si4A	0.515 (3)	0.5685 (12)	0.7787 (8)	0.1318 (13)	0.375 (9)
C18	0.445 (3)	0.639 (2)	0.8231 (9)	0.161 (3)	0.375 (9)
H18D	0.4651	0.6110	0.8550	0.242*	0.375 (9)
H18E	0.3448	0.6441	0.8186	0.242*	0.375 (9)
H18F	0.4868	0.7069	0.8204	0.242*	0.375 (9)
C20	0.432 (3)	0.4260 (15)	0.7663 (10)	0.170 (3)	0.375 (9)
H20D	0.4911	0.3823	0.7465	0.255*	0.375 (9)
H20E	0.3431	0.4366	0.7502	0.255*	0.375 (9)
H20F	0.4164	0.3934	0.7975	0.255*	0.375 (9)
C19A	0.480 (3)	0.526 (3)	0.7151 (9)	0.151 (3)	0.375 (9)
H19D	0.5197	0.5750	0.6926	0.226*	0.375 (9)
H19E	0.3795	0.5215	0.7100	0.226*	0.375 (9)
H19F	0.5217	0.4589	0.7098	0.226*	0.375 (9)

### Atomic displacement parameters (Å^2^)

**Table:**

	*U*^11^	*U*^22^	*U*^33^	*U*^12^	*U*^13^	*U*^23^
Si1	0.0804 (9)	0.0542 (8)	0.0802 (9)	−0.0045 (7)	0.0034 (8)	0.0013 (8)
Si2	0.1054 (12)	0.0709 (10)	0.0970 (12)	0.0005 (9)	−0.0386 (10)	0.0153 (9)
Si3	0.1152 (12)	0.0534 (8)	0.0839 (10)	−0.0089 (9)	−0.0044 (10)	−0.0025 (8)
Si4	0.1162 (18)	0.180 (3)	0.0987 (19)	0.026 (3)	−0.0233 (15)	−0.043 (2)
O1	0.078 (2)	0.058 (2)	0.082 (2)	0.0030 (19)	−0.0269 (19)	0.001 (2)
O2	0.0583 (17)	0.0556 (19)	0.085 (2)	−0.0022 (15)	−0.0030 (16)	−0.0010 (17)
O3	0.0407 (19)	0.177 (4)	0.129 (4)	0.011 (2)	0.009 (2)	−0.036 (3)
O4	0.0725 (19)	0.068 (2)	0.079 (2)	−0.0113 (19)	−0.0134 (18)	0.0178 (19)
O5	0.087 (2)	0.0523 (18)	0.086 (2)	0.0013 (19)	−0.0010 (19)	0.0020 (18)
O6	0.130 (3)	0.094 (3)	0.111 (3)	−0.025 (3)	−0.054 (3)	0.004 (3)
N1	0.0321 (15)	0.079 (3)	0.070 (3)	0.0023 (19)	0.0012 (18)	−0.009 (2)
C1	0.054 (3)	0.059 (3)	0.080 (4)	0.006 (2)	−0.008 (2)	−0.004 (3)
C2	0.038 (2)	0.069 (3)	0.067 (3)	−0.001 (2)	−0.005 (2)	0.000 (3)
C3	0.048 (2)	0.058 (3)	0.072 (3)	−0.005 (2)	−0.005 (2)	0.010 (3)
C4	0.064 (3)	0.052 (3)	0.067 (3)	−0.008 (2)	−0.009 (2)	0.000 (3)
C5	0.076 (3)	0.057 (3)	0.074 (3)	−0.001 (3)	−0.008 (3)	0.000 (3)
C6	0.158 (6)	0.078 (4)	0.104 (5)	−0.008 (4)	0.023 (5)	−0.022 (4)
C7	0.100 (4)	0.096 (5)	0.095 (4)	−0.015 (4)	−0.003 (4)	0.010 (4)
C8	0.112 (5)	0.074 (4)	0.153 (7)	0.011 (4)	−0.012 (4)	0.027 (5)
C9	0.047 (3)	0.088 (4)	0.080 (4)	0.002 (3)	0.005 (3)	0.004 (3)
C10	0.073 (3)	0.116 (5)	0.074 (4)	0.005 (4)	0.008 (3)	−0.014 (4)
C11	0.167 (7)	0.158 (7)	0.093 (5)	0.037 (6)	−0.053 (5)	0.003 (5)
C12	0.099 (5)	0.118 (6)	0.172 (7)	0.030 (5)	−0.046 (5)	0.004 (6)
C13	0.186 (8)	0.117 (6)	0.186 (9)	−0.038 (6)	−0.079 (7)	0.073 (6)
C14	0.157 (6)	0.056 (4)	0.183 (9)	0.009 (4)	−0.006 (6)	−0.007 (5)
C15	0.148 (6)	0.089 (5)	0.134 (6)	−0.038 (5)	0.013 (5)	−0.015 (5)
C16	0.244 (10)	0.087 (5)	0.089 (5)	−0.013 (6)	−0.029 (6)	−0.012 (4)
C17	0.128 (5)	0.076 (4)	0.072 (4)	−0.005 (4)	−0.026 (4)	0.002 (3)
C18A	0.143 (5)	0.204 (7)	0.137 (6)	0.032 (5)	−0.018 (5)	−0.040 (5)
C19	0.128 (5)	0.202 (7)	0.122 (5)	0.031 (6)	−0.036 (4)	−0.032 (6)
C20A	0.145 (5)	0.218 (7)	0.148 (6)	0.008 (6)	−0.015 (5)	−0.032 (6)
Si4A	0.1162 (18)	0.180 (3)	0.0987 (19)	0.026 (3)	−0.0233 (15)	−0.043 (2)
C18	0.143 (5)	0.204 (7)	0.137 (6)	0.032 (5)	−0.018 (5)	−0.040 (5)
C20	0.145 (5)	0.218 (7)	0.148 (6)	0.008 (6)	−0.015 (5)	−0.032 (6)
C19A	0.128 (5)	0.202 (7)	0.122 (5)	0.031 (6)	−0.036 (4)	−0.032 (6)

### Geometric parameters (Å, º)

**Table:**

Si1—O2	1.649 (4)	C9—C10	1.498 (8)
Si1—C7	1.833 (6)	C10—H10A	0.9600
Si1—C8	1.845 (7)	C10—H10B	0.9600
Si1—C6	1.847 (6)	C10—H10C	0.9600
Si2—O4	1.644 (4)	C11—H11A	0.9600
Si2—C12	1.848 (8)	C11—H11B	0.9600
Si2—C13	1.851 (8)	C11—H11C	0.9600
Si2—C11	1.863 (7)	C12—H12A	0.9600
Si3—O5	1.655 (4)	C12—H12B	0.9600
Si3—C15	1.833 (7)	C12—H12C	0.9600
Si3—C14	1.851 (8)	C13—H13A	0.9600
Si3—C16	1.886 (7)	C13—H13B	0.9600
Si4—O6	1.60 (2)	C13—H13C	0.9600
Si4—C18	1.846 (10)	C14—H14A	0.9600
Si4—C19	1.850 (9)	C14—H14B	0.9600
Si4—C20	1.858 (10)	C14—H14C	0.9600
O1—C1	1.408 (6)	C15—H15A	0.9600
O1—C5	1.432 (6)	C15—H15B	0.9600
O2—C1	1.406 (6)	C15—H15C	0.9600
O3—C9	1.224 (5)	C16—H16A	0.9600
O4—C3	1.427 (5)	C16—H16B	0.9600
O5—C4	1.419 (6)	C16—H16C	0.9600
O6—C17	1.396 (7)	C17—H17A	0.9700
O6—Si4A	1.69 (3)	C17—H17B	0.9700
N1—C9	1.317 (6)	C18A—Si4A	1.856 (10)
N1—C2	1.447 (6)	C18A—H18A	0.9600
N1—H1	0.8600	C18A—H18B	0.9600
C1—C2	1.532 (7)	C18A—H18C	0.9600
C1—H1A	0.9800	C19—H19A	0.9600
C2—C3	1.533 (6)	C19—H19B	0.9600
C2—H2	0.9800	C19—H19C	0.9600
C3—C4	1.505 (6)	C20A—Si4A	1.856 (10)
C3—H3	0.9800	C20A—H20A	0.9600
C4—C5	1.525 (7)	C20A—H20B	0.9600
C4—H4	0.9800	C20A—H20C	0.9600
C5—C17	1.511 (7)	Si4A—C19A	1.851 (10)
C5—H5	0.9800	C18—H18D	0.9600
C6—H6A	0.9600	C18—H18E	0.9600
C6—H6B	0.9600	C18—H18F	0.9600
C6—H6C	0.9600	C20—H20D	0.9600
C7—H7A	0.9600	C20—H20E	0.9600
C7—H7B	0.9600	C20—H20F	0.9600
C7—H7C	0.9600	C19A—H19D	0.9600
C8—H8A	0.9600	C19A—H19E	0.9600
C8—H8B	0.9600	C19A—H19F	0.9600
C8—H8C	0.9600		
			
O2—Si1—C7	105.4 (3)	Si1—C8—H8C	109.5
O2—Si1—C8	108.8 (3)	H8A—C8—H8C	109.5
C7—Si1—C8	111.8 (3)	H8B—C8—H8C	109.5
O2—Si1—C6	109.6 (3)	O3—C9—N1	121.3 (5)
C7—Si1—C6	111.1 (3)	O3—C9—C10	121.0 (5)
C8—Si1—C6	110.1 (4)	N1—C9—C10	117.7 (4)
O4—Si2—C12	110.0 (3)	C9—C10—H10A	109.5
O4—Si2—C13	105.8 (3)	C9—C10—H10B	109.5
C12—Si2—C13	109.4 (4)	H10A—C10—H10B	109.5
O4—Si2—C11	112.8 (3)	C9—C10—H10C	109.5
C12—Si2—C11	106.9 (4)	H10A—C10—H10C	109.5
C13—Si2—C11	111.9 (5)	H10B—C10—H10C	109.5
O5—Si3—C15	111.2 (3)	Si2—C11—H11A	109.5
O5—Si3—C14	105.2 (3)	Si2—C11—H11B	109.5
C15—Si3—C14	113.1 (4)	H11A—C11—H11B	109.5
O5—Si3—C16	111.0 (3)	Si2—C11—H11C	109.5
C15—Si3—C16	108.0 (4)	H11A—C11—H11C	109.5
C14—Si3—C16	108.3 (4)	H11B—C11—H11C	109.5
O6—Si4—C18	109.0 (12)	Si2—C12—H12A	109.5
O6—Si4—C19	112.4 (9)	Si2—C12—H12B	109.5
C18—Si4—C19	121.8 (15)	H12A—C12—H12B	109.5
O6—Si4—C20	101.9 (11)	Si2—C12—H12C	109.5
C18—Si4—C20	116.8 (15)	H12A—C12—H12C	109.5
C19—Si4—C20	92.6 (13)	H12B—C12—H12C	109.5
C1—O1—C5	114.0 (3)	Si2—C13—H13A	109.5
C1—O2—Si1	124.1 (3)	Si2—C13—H13B	109.5
C3—O4—Si2	130.0 (3)	H13A—C13—H13B	109.5
C4—O5—Si3	129.2 (3)	Si2—C13—H13C	109.5
C17—O6—Si4	130.2 (5)	H13A—C13—H13C	109.5
C17—O6—Si4A	126.2 (7)	H13B—C13—H13C	109.5
C9—N1—C2	123.7 (4)	Si3—C14—H14A	109.5
C9—N1—H1	118.2	Si3—C14—H14B	109.5
C2—N1—H1	118.2	H14A—C14—H14B	109.5
O2—C1—O1	110.9 (4)	Si3—C14—H14C	109.5
O2—C1—C2	109.5 (3)	H14A—C14—H14C	109.5
O1—C1—C2	110.8 (4)	H14B—C14—H14C	109.5
O2—C1—H1A	108.5	Si3—C15—H15A	109.5
O1—C1—H1A	108.5	Si3—C15—H15B	109.5
C2—C1—H1A	108.5	H15A—C15—H15B	109.5
N1—C2—C1	110.3 (4)	Si3—C15—H15C	109.5
N1—C2—C3	113.1 (3)	H15A—C15—H15C	109.5
C1—C2—C3	110.9 (4)	H15B—C15—H15C	109.5
N1—C2—H2	107.4	Si3—C16—H16A	109.5
C1—C2—H2	107.4	Si3—C16—H16B	109.5
C3—C2—H2	107.4	H16A—C16—H16B	109.5
O4—C3—C4	109.2 (4)	Si3—C16—H16C	109.5
O4—C3—C2	108.7 (4)	H16A—C16—H16C	109.5
C4—C3—C2	110.3 (4)	H16B—C16—H16C	109.5
O4—C3—H3	109.5	O6—C17—C5	113.3 (5)
C4—C3—H3	109.5	O6—C17—H17A	108.9
C2—C3—H3	109.5	C5—C17—H17A	108.9
O5—C4—C3	109.6 (4)	O6—C17—H17B	108.9
O5—C4—C5	108.6 (4)	C5—C17—H17B	108.9
C3—C4—C5	111.6 (4)	H17A—C17—H17B	107.7
O5—C4—H4	109.0	Si4A—C18A—H18A	109.5
C3—C4—H4	109.0	Si4A—C18A—H18B	109.5
C5—C4—H4	109.0	H18A—C18A—H18B	109.5
O1—C5—C17	106.4 (4)	Si4A—C18A—H18C	109.5
O1—C5—C4	109.9 (4)	H18A—C18A—H18C	109.5
C17—C5—C4	113.4 (4)	H18B—C18A—H18C	109.5
O1—C5—H5	109.0	Si4A—C20A—H20A	109.5
C17—C5—H5	109.0	Si4A—C20A—H20B	109.5
C4—C5—H5	109.0	H20A—C20A—H20B	109.5
Si1—C6—H6A	109.5	Si4A—C20A—H20C	109.5
Si1—C6—H6B	109.5	H20A—C20A—H20C	109.5
H6A—C6—H6B	109.5	H20B—C20A—H20C	109.5
Si1—C6—H6C	109.5	O6—Si4A—C19A	104.8 (14)
H6A—C6—H6C	109.5	O6—Si4A—C18A	113.3 (14)
H6B—C6—H6C	109.5	C19A—Si4A—C18A	118.8 (17)
Si1—C7—H7A	109.5	O6—Si4A—C20A	105.7 (12)
Si1—C7—H7B	109.5	C19A—Si4A—C20A	95.6 (16)
H7A—C7—H7B	109.5	C18A—Si4A—C20A	116.5 (14)
Si1—C7—H7C	109.5	Si4A—C19A—H19D	109.5
H7A—C7—H7C	109.5	Si4A—C19A—H19E	109.5
H7B—C7—H7C	109.5	H19D—C19A—H19E	109.5
Si1—C8—H8A	109.5	Si4A—C19A—H19F	109.5
Si1—C8—H8B	109.5	H19D—C19A—H19F	109.5
H8A—C8—H8B	109.5	H19E—C19A—H19F	109.5

### Hydrogen-bond geometry (Å, º)

**Table:**

*D*—H···*A*	*D*—H	H···*A*	*D*···*A*	*D*—H···*A*
N1—H1···O3^i^	0.86	2.03	2.855 (4)	160

Symmetry code: (i) *x*+1/2, −*y*+3/2, −*z*+2.
